# Bisphosphonates do not affect healing of a critical-size defect in estrogen-deficient mice

**DOI:** 10.1016/j.bonr.2024.101739

**Published:** 2024-01-17

**Authors:** Franziska Strunz, Saskia Gentil-Perret, Mark Siegrist, Marc Bohner, Nikola Saulacic, Willy Hofstetter

**Affiliations:** a Bone & Joint Program, Department for BioMedical Research (DBMR), University of Bern, Bern, Switzerland; b Graduate School for Cellular and Biomedical Sciences, University of Bern, Switzerland; c RMS-Foundation, Bettlach, Switzerland; d Clinic for Cranio-Maxillofacial Surgery, Department for BioMedical Research (DBMR), University of Bern, Bern, Switzerland; e Cardiovascular Diseases Program, Department for BioMedical Research, University of Bern, Bern, Switzerland

**Keywords:** Osteoporosis, Bisphosphonate, Critical-size defect, β-tri-calcium-phosphate, Bone morphogenetic protein-2, Bone repair, BMP antagonist

## Abstract

Bisphosphonates (BP) are anti-resorptive drugs that are widely used to prevent bone loss in osteoporosis. Since inhibition of bone resorption will cause a decrease in bone formation through a process called coupling, it is hypothesized that extended treatment protocols may impair bone healing. In this study, β-tri‑calcium-phosphate (βTCP) ceramics were inserted into critical-size long bone defects in estrogen-deficient mice under BP therapy. The study assessed the benefits of coating the ceramics with Bone Morphogenetic Protein-2 (BMP2) and an engineered BMP2 analogue (L51P) that inactivates BMP antagonists on the healing process, implant resorption, and bone formation.

Female NMRI mice (11–12 weeks of age) were ovariectomized (*OVX*) or sham operated. Eight weeks later, after the manifestation of ovariectomy-induced osteoporotic bone changes, BP therapy with Alendronate (ALN) was commenced. After another five weeks, a femoral critical-size defect was generated, rigidly fixed, and βTCP-cylinders loaded with 0.25 μg or 2.5 μg BMP2, 2.5 μg L51P, and 0.25 μg BMP2/2.5 μg L51P, respectively, were inserted. Unloaded βTCP-cylinders were used as controls. Femora were collected six and twelve weeks post-implantation.

Histological and micro-computer tomography (MicroCT) evaluation revealed that insertion of cylinders coated with 2.5 μg BMP2 accelerated fracture repair and induced significant bone formation compared to controls (unloaded cylinders or coated with 2.5 μg L51P, 0.25 μg BMP2) already six weeks post-implantation, independent of estrogen-deficiency and BP therapy. The simultaneous administration of BMP2 and L51P (0.25 μg BMP2/2.5 μg L51P) did not promote fracture healing six and twelve weeks post-implantation. Moreover, new bone formation within the critical-size defect was directly linked to the removal of the βTCP-implant in all experimental groups. No evidence was found that long-term therapy with ALN impaired the resorption of the implanted graft. However, osteoclast transcriptome signature was elevated in sham and *OVX* animals upon treatment with BP, with transcript levels being higher at six weeks than at twelve weeks post-surgery. Furthermore, the transcriptome profile of the developing repair tissue confirmed an accelerated repair process in animals treated with 2.5 μg BMP2 implants. L51P did not increase the bioefficacy of BMP2 in the applied defect model.

The present study provides evidence that continuous administration of BP does not inhibit implant resorption and does not alter the kinetics of the healing process of critical-size long bone defects. Furthermore, the BMP2 variant L51P did not enhance the bioefficacy of BMP2 when applied simultaneously to the femoral critical-size defect in sham and *OVX* mice.

## Highlights

• Resorption of βTCP ceramic implants and formation of new bone are coupled during the repair of a critical-size bone defect.
• Therapy with the bisphosphonate Alendronate does not alter the kinetics of the healing of a femoral critical-size defect in estrogen-deficient mice.
• BMP2 improves healing of a femoral critical-size defect and the variant L51P does not enhance the bioefficacy of BMP2 in the applied model.

## 1 Introduction

Osteoporosis is a major health issue in aging societies and affects hundreds of millions of people worldwide, approx. 80 % among them post-menopausal women (Willers et al., 2022). Dysregulated bone turnover with a negative remodeling balance results in loss of bone mass and microarchitectural deterioration, leading to increased fragility and fracture risk (Zebaze et al., 2010; Parfitt et al., 1983; Compston et al., 2019; Liu et al., 2019).

Bisphosphonates (BP) are the most frequent therapy to prevent bone loss in post-menopausal osteoporosis (Liu et al., 2019; Bone et al., 1997; Cranney et al., 2002; Hosking et al., 1998; Liberman et al., 1995). BP bind with high affinity to bone mineral and exhibit long-term action on bone metabolism (Rodan and Fleisch, 1996; Russell, 2006). Bone turnover is efficiently attenuated by BP since the suppression of osteoclastic bone resorption upon cellular uptake of the drugs will lead, through a coupling mechanism, to a decrease in bone formation (Luckman et al., 1998; Weinstein et al., 2009; Hughes et al., 1995). This “frozen” bone state in extended treatment protocols may impair bone healing in elderly patients that are prone to fractures (Diab and Watts, 2012; McClung et al., 2013; Watts and Diab, 2010; Odvina et al., 2005; Molvik and Khan, 2015). Furthermore, large bone defects frequently require the implantation of bone grafts, such as calcium phosphate-based ceramics like β-tricalcium phosphate (β-Ca_3_(PO_4_)_2_, βTCP) (Galois et al., 2002; Chazono et al., 2004; Bohner, 2000). The manufacturing process of βTCP grafts enables the modification of pore size and connectivity, mechanical stability, and porosity. This allows optimization of the biomaterial to support the invasion by bone cell lineages and stimulate angiogenesis (Bohner et al., 2020; Malhotra and Habibovic, 2016). Essentially, calcium phosphate-based materials are already approved for clinical application and widely used in orthopedic surgery and dentistry (Dorozhkin, 2010; Rolvien et al., 2017; Roca-Millan et al., 2022; Tanaka et al., 2017; Guillaume, 2017).

The replacement of grafted materials by newly formed bone is essential to ensure the restoration of the mechanical stability of the damaged site. BP inhibit osteoclast-mediated bone resorption, and possibly block implant resorption. Various *in vivo* approaches studied the effects of BP on fracture healing in rodents and reported varying outcomes, ranging from delayed bone healing to superior mechanical stability of the defect tissue (Gerstenfeld et al., 2009; Hauser et al., 2018a; Hadjiargyrou, 2022). However, in several rat studies, delayed bone unions and impaired healing were observed in the presence of BP (Li et al., 2001; Li et al., 1999; Kidd et al., 2011; Manabe et al., 2012). In particular, ALN treatment caused delayed bone remodeling and implant removal, as well as increased callus sizes in rats (Cao et al., 2002; Fu et al., 2013; Hauser et al., 2018b).

Since the capacity of bone to heal decreases with age, bone grafts were combined with osteoinductive growth factors to support bone regeneration (Roddy et al., 2018; Schmidmaier et al., 2009; Newman and Benoit, 2016). Recombinant human Bone morphogenetic proteins (BMP) like BMP2 are approved for the treatment of non-healing defects and the stimulation of bone fusions (Lowery and Rosen, 2018; Gillman and Jayasuriya, 2021; Gautschi et al., 2007). The spatial and temporal availability of the administered BMP2 might not be optimal to support the healing process and the upregulation of BMP antagonists can further decrease the bioefficacy of the applied growth factor. To enhance the bioavailability of exogenously administered BMP2, supraphysiological doses are used in clinical procedures to compensate for the short action of the growth factor owing to the fast release kinetics and diffusion (Mesfin et al., 2013; Canalis et al., 2003; Dean et al., 2010). Increasing concentrations of the applied BMP2, thus, did not always lead to sufficient bone formation and were even associated with inflammatory responses, ectopic bone formation, and osteolysis (James et al., 2016; Carragee et al., 2011; Nguyen et al., 2017).

A possible strategy to overcome the limitations of the low bioefficacy of BMP2 is presented by the synthetic BMP2 variant L51P, with a leucine to proline substitution at amino acid position 51 interrupting the major binding site with the BMP receptor type I. L51P exhibits high binding affinity to BMP antagonists like noggin, chordin and gremlin without the activation of BMP signaling (Keller et al., 2004). L51P efficiently blocked BMP antagonists *in vitro* (Keller et al., 2004; Albers et al., 2012; Khattab et al., 2014) and enhanced the bioactivity of BMP2 *in vivo* (Hauser et al., 2018b; Khattab et al., 2014; Sebald et al., 2012; Khattab et al., 2019). Moreover, L51P reduced the necessary amount of BMP2 to induce bone formation in a rat calvaria defect model (Khattab et al., 2014) and in critical-size defects fitted with βTCP ceramic implants (Hauser et al., 2018b; Sebald et al., 2012).

The present study aimed to evaluate implant resorption and bone formation during the healing process of a femoral critical-size defect in estrogen-deficient mice fitted with βTCP-ceramics. The bioefficacy of BMP2, combined with the BMP variant L51P, with BP therapy and estrogen-depletion, was assessed.

## 2 Methods

### 2.1 Design of the animal study

This study was performed in accordance with Swiss Federal regulations and approved by the Cantonal Veterinary Office (permit numbers BE86/18 and BE84/21 to WH). Animals were kept under specific pathogen-free conditions in the Central Animal Facility of the University of Bern. Female outbred NMRI (Han) mice (11–12 weeks, Charles River, Sulzfeld, GER) were assigned to one of the 20 experimental groups representing the combinations of surgical procedures, treatments, and two time points: sham/ovariectomy (*OVX*), Vehicle (Veh)/Alendronate (ALN) therapy, implant coating (0 μg BMP2/0 μg L51P; 2.5 μg L51P; 0.25 μg BMP2; 0.25 μg BMP2/2.5 μg L51P; 2.5 μg BMP2). Samples were collected six and twelve weeks after application of the femoral critical-size defects.

**Fig. 1:**
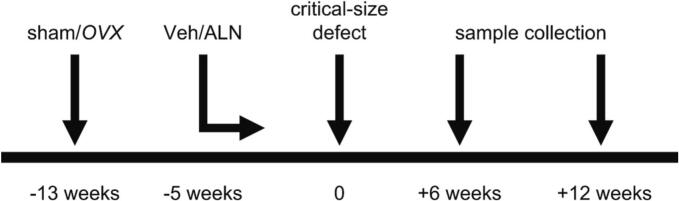
Experimental design of the ALN-treated ovariectomy-induced osteoporotic femoral defect model.

The experimental setup of the study is depicted in Fig. 1. Briefly, animals underwent either *OVX* or sham surgery (Hauser et al., 2018a). Veh/ALN treatment was initiated eight weeks later, and continued until sacrifice of the animals (Sato et al., 1991). Five weeks after the onset of Veh/ALN treatment, a critical-size defect (3.5 mm) was applied in the left femur of all animals. The defect was filled with βTCP-cylinders loaded with 2.5 μg L51P, 0.25 μg BMP2, 0.25 μg BMP2/2.5 μg L51P, 2.5 μg BMP2 or unloaded controls (0 μg BMP2/0 μg L51P). The defect site was rigidly fixed using a titanium osteosynthesis system (MouseFix™ plate 6 hole, RIS.401.130; RISystem AG, Davos, CH). Animals were sacrificed six and twelve weeks after setting the defect. Groups for histology and MicroCT included 2–5 animals (101 and 105 animals in total, respectively), and for RNA sequencing 3 animals (96 animals in total). For the time point six weeks, only the coatings 0.25 μg BMP2, 0.25 μg BMP2/2.5 μg L51P, and 2.5 μg BMP2 were used.

### 2.2 Anesthesia and analgesia during surgery and post-surgical treatment

Body weight was assessed before *OVX*/sham surgery and throughout the study. Before surgical procedures, animals were anesthetized by intraperitoneal (*i.p.*) injections (2 ml/kg body weight) with a mixture of Medetomidine (0.5 mg/kg; Dorbene®, Dr. E. Graeub AG, Bern, CH), Midazolam (5 mg/kg; Dormicum®, Roche, Basel, CH), and Fentanyl (0.05 mg/kg; Fentanyl-Mepha®, Mepha, Basel, CH). Post-operatively, an antidote composed of Atipamezole (0.23 mg/kg; Alzane®, Dr. E. Graeub AG), Flumazenil (0.25 mg/kg; Anexate®, Roche), and Buprenorphin (0.075 mg/kg; Bupaq®, Streuli Pharma AG, Uznach, CH) was applied subcutaneously (*s.c.*) (2.5 ml/kg body weight). Analgesia (Buprenorphin, 0.075 mg/kg, *s.c.*) was administered before the sham/*OVX* and the femoral defect surgeries and 24 or 72 h after the operations, respectively.

### 2.3 Ovariectomy

The removal of ovaries (*OVX*) to generate the estrogen-deficient mouse model for postmenopausal osteoporosis was performed as previously described (Hauser et al., 2018a). Briefly, two 0.5 cm flank incisions at the mid-dorsum were applied to locate the ovaries. During the *OVX* procedure, the oviducts were ligated with an absorbable thread (Coated VICRYL® 5-0, Ethicon, Zug, CH), and the ovaries were removed. Subsequently, the peritoneum was closed with absorbable sutures, and the skin with non-absorbable polypropylene threads (PROLENETM 5-0, Ethicon). Sham animals underwent the identical surgical procedure without ligation of the oviducts and removal of the ovaries. At the end of the study, uteri were collected, and uterus dry weight was assessed after drying in air for 24 h.

### 2.4 Treatment with Alendronate

Alendronate (ALN; Sigma-Aldrich, Buchs, CH) was dissolved in 0.9 % NaCl and sterile filtered (Sato et al., 1991). Experimental groups with anti-resorptive therapy received *s.c.* ALN injections (1.61 mmol/kg body weight; 0.2 ml/100 g body weight) twice a week, starting eight weeks after the sham or *OVX* operation, until sacrifice. Control animals received Veh solution (0.9 % NaCl solution; 0.2 ml/100 g body weight).

### 2.5 Loading of βTCP-cylinders

The critical-size defect was filled with βTCP-cylinders with a diameter of 2.5 mm, a length of 3.5 mm, and a porosity of 60 % (Robert Mathys Foundation, Bettlach, CH) (von Doernberg et al., 2006; Bohner et al., 2017; Bohner et al., 2005). Before implantation, the cylinders were coated with growth factors: 2.5 μg L51P, 0.25 μg BMP2, 0.25 μg BMP2/2.5 μg L51P, 2.5 μg BMP2 or unloaded (0 μg BMP2/0 μg L51P). The human recombinant BMP2 and L51P proteins were expressed in *Escherichia coli* and were kindly provided by Prof. W Sebald (University of Würzburg, GE).

### 2.6 Femoral critical-size defect

The critical-size defect was created in the left femur of all animals 13 weeks after sham/*OVX* surgery and five weeks after the onset of Veh/ALN therapy, as described previously (Manassero et al., 2012). After anesthesia, the skin was shaved and disinfected, and a longitudinal incision was made into the left thigh parallel to the femur. In the next step, a six-hole titanium plate developed for osteosynthesis in murine models was mounted onto the femur with four interlocking screws into the two outer plate holes on both sides. Subsequently, to create a reproducible mid-femoral critical-size defect (3.5 mm), a self-constructed guiding tool was fixed to the two inner screws. Two Gigli saws were used to cut two osteotomies, and the defect site was rinsed with sterile physiological saline solution. To bridge the bone defect, βTCP-cylinders with varying coatings of BMP2 and L51P were press-fit into the defects and fixed with a non-absorbable thread. Subsequently, the thigh muscle tissue was realigned and stitched with an absorbable thread, and the wound was closed with non-absorbable sutures. To support the recovery from the surgery and the anesthesia, an antidote mixture was injected, and analgesia was applied for 72 h. Animals were monitored daily according to a pre-defined score sheet. Load bearing or movement was not restricted after surgery.

The integrity of the surgical site and the placement of the βTCP-implant were examined post-operatively by high-resolution radiography (MX-20, Faxitron X-Ray Corporation, Edimex, Le Plessis, FR) (Suppl. Fig. 1). In total, 260 animals underwent surgery; 17 mice were excluded from the analysis because of incorrect plate or implant positioning, and five animals were euthanized due to health issues independent of the surgical protocol.

### 2.7 MicroCT

Tissues for MicroCT analysis and histology were fixed in 4 % paraformaldehyde in phosphate-buffered saline for 24 h, rinsed with tap water, and transferred to 70 % ethanol. Femora with a critical-size defect were examined by MicroCT analysis (MicroCT40, SCANCO Medical AG, Brüttisellen, CH), by placing the long axis of the bone orthogonally to the axis of the X-ray beam (Bouxsein et al., 2010). The X-ray tube was operated at 70 kVp and 57 μA with an integration time of 300 ms. The analysis was performed at a resolution of 8 μm. The composition of the defect tissue, including the total volume of the repair tissue, the volume of mineralized tissue consisting of newly formed bone and remaining implant material, and the bone volume/total volume (BV/TV) was assessed using the built-in software from Scanco (Scanco Module 64-bit; V5.15).

The examination of vertebrae was carried out with identical settings of the X-ray tube at a resolution of 6 μm. The measurements were recorded perpendicularly to the longitudinal axis of the vertebrae. The region of interest in the center of the vertebral body of the lumbar vertebra four (L4) was defined manually by using the built-in software from Scanco.

### 2.8 Histology and histomorphometry

For histomorphometric analysis, tissue samples were embedded in methyl methacrylate as described previously (Wernike et al., 2010). Ground sections of approx. 600–800 μm were cut with a diamond saw (Leco VC-50, Leco Corporation, St. Joseph, Michigan, USA). After grinding to a thickness of approx. 300 μm and polishing, sections were stained with toluidine blue mixed with MacNeal's tetrachrome solution and fuchsin red (0.05 %). Images were captured with a digital microscope (VHX-6000, Keyence, Mechelen, BE). The quantification of the remaining implant material and the newly formed bone in the defect site was done by manual selection (Photoshop, version 24.0) based on color gradients (Dempster et al., 2013).

### 2.9 RNA sequencing

#### 2.9.1 Isolation of total RNA

For RNA extraction and library preparation, the tissue between the two inner central screws containing the remaining implant material and newly formed bone tissue was dissected, placed in RNALater® (Sigma-Aldrich), and stored at −20 °C. For total RNA extraction, samples were transferred into microtubes containing 1 ml TRIzol™ Reagent (Invitrogen™) and metal beads for tissue disruption in a benchtop tissue homogenizer (TissueLyser 2, Qiagen, Hilden, DE).

RNA was isolated according to the manufacturer's instructions using the NucleoSpin® RNA Plus Kit (Macherey-Nagel, Oensingen, CH), and genomic DNA was removed by digestion with the provided DNase. The quantity and quality of the purified total RNA was assessed using a Thermo Fisher Scientific Qubit 4.0 fluorometer with the Qubit RNA BR Assay Kit (Q10211, Thermo Fisher Scientific, Waltham, Massachusetts, USA) and an Advanced Analytical Fragment Analyzer System using a Fragment Analyzer RNA Kit (DNF-471, Agilent Technologies, Santa Clara, California, USA), respectively.

#### 2.9.2 Preparation of libraries, sequencing, and mapping to reference genome

In total, 32 experimental groups with 3 biological replicates (96 samples in total) each underwent RNA sequencing. Sequencing libraries were prepared with 500 ng input RNA using an Illumina TruSeq Stranded mRNA Library Prep kit (20020595, Illumina, San Diego, California, USA) in combination with TruSeq RNA UD Indexes (20022371, Illumina) according to the manufacturer's guidelines. Pooled cDNA libraries were sequenced paired-end using a shared Illumina NovaSeq 6000 S4 Reagent Kit (300 cycles; 20028312, Ilumina) on an Illumina NovaSeq 6000 instrument. This run was performed in the NovaSeq Xp workflow using a NovaSeq XP 4-Lane Kit v1.5 (20043131, Illumina). On average, the run produced 32.7 million reads/library. The quality of the sequencing run was assessed using Illumina Sequencing Analysis Viewer (lllumina version 2.4.7), and all base call files were demultiplexed and converted into FASTQ files using Illumina bcl2fastq conversion software v2.20. The quality control assessments, generation of libraries, and sequencing were conducted by the Next Generation Sequencing Platform of the University of Bern. The quality of the sequencing data was assessed by FastQC version (v.) 0.11.9 (*Babraham Bioinformatics - FastQC A Quality Control tool for High Throughput Sequence Data*, n.d.). The reference genome (assembly GRCm39) and associated annotation were obtained from the Ensembl genome database (*Ensembl genome browser* 109, n.d.). The reference genome was indexed using STAR (v. 2.7.10) (Dobin et al., 2013). STAR was also used to calculate the table of counts containing the number of reads per gene.

#### 2.9.3 Differentially expressed genes and GO enrichment analysis

Principal component analysis was performed based on the 500 genes with the most variable expression over the whole transcriptome to reveal differences with respect to estrogen-deficiency, BP therapy, time point after implantation, and the availability of BMP2 and L51P. The differential gene expression analyses to determine the effects of BP therapy, estrogen-deficiency, and implant coating were carried out using the Bioconductor package DESeq2 (v. 1.32.0) (Love et al., 2014) in R (v. 4.1.0) (*R: The R Project for Statistical Computing*, n.d.). To increase stringency, only differently expressed genes (DEG) with a two-fold differential expression were considered for further analysis. Differences in expression with a false discovery rate smaller than 0.05 were considered significant. Gene ontology (GO) enrichment analysis was performed when >100 genes were significantly differentially expressed. To identify GO terms, the enrichGO function from the clusterProfiler package (v. 4.0.5) (Wu et al., 2021) was used, along with the Bioconductor package org.Mm.eg.db (Carlson, n.d.) containing the genome-wide annotation for mouse. Changes in transcript levels encoding osteoclast and osteoblast traits with respect to BP therapy and time points six and twelve weeks after application of the defect were investigated. After removing the dependence of the variance on the mean using DESeq2::vst(), heatmaps of selected marker genes were produced using pheatmap (v. 1.0.12) (Kolde, 2019).

### 2.10 Statistical analysis

The statistical analyses were performed using GraphPad Prism 9 for Windows (GraphPad Software, San Diego, California, USA). The unpaired *t*-test was used for the analysis of body weight and uterus dry weights, and one-way or two-way analysis of variance (ANOVA) with Tukey post-hoc was applied to analyze MicroCT and histomorphometric data. *P* values smaller than 0.05 were considered significant.

## 3 Results

### 3.1 Characterization of the post-menopausal osteoporosis model and the effects of BP therapy

The suitability of the murine post-menopausal osteoporosis model in NMRI mice was assessed by analyzing body weight, uterus dry weight, and bone architecture upon estrogen-deficiency. The average body weight was significantly increased in *OVX* animals at 13, 19, and 25 weeks after *OVX* surgery when compared to sham animals (*p* < 0.01) (Suppl. Fig. 2a). The average uterus dry weight of *OVX* animals was significantly lower compared to sham controls (*p* < 0.001) (Suppl. Fig. 2b).

The effects of *OVX* and ALN therapy on bone mass and structure were assessed by MicroCT analysis of the vertebral body of L4 in samples harvested 19 and 25 weeks after OVX/sham surgery, equivalent to 11 and 17 weeks after the onset of BP/Veh therapy, respectively (Suppl. Fig. 3). The BV/TV of L4 was decreased by 32.4 ± 26.0 % (*p* < 0.01) in *OVX*/Veh animals when compared to sham/Veh controls. ALN therapy increased the BV/TV on average by 55.3 ± 35.0 % (p < 0.01) in the *OVX*/ALN animals as compared to the *OVX*/Veh group. In sham/ALN animals, the BV/TV was 56.1 ± 22.8 % (*p* < 0.001) higher than in the sham/Veh group. BP therapy preserved bone mass and structure in the vertebral body of L4 upon estrogen-depletion. Treatment with ALN led to significantly higher BV/TV in both sham and *OVX* animals, a significant difference, however, remained between the sham/ALN and *OVX*/ALN groups, with lower values in estrogen-deficient animals.

### 3.2 Bone healing process in the femoral critical-size defects

For the time point of six weeks post-surgery, implant coatings 0.25 μg BMP2, 0.25 μg BMP2/2.5 μg L51P and 2.5 μg BMP2 were included in the experimental design and loading with 0.25 μg BMP2 was used as the negative control. For the time point twelve weeks post-surgery, all implant coatings were included in the evaluation of bone formation and implant removal by histology and MicroCT analysis. Unloaded cylinders together with implants coated with 2.5 μg L51P and 0.25 μg BMP2 were used as negative controls to assess the induction of bone growth by 2.5 μg BMP2 and 0.25 μg BMP2/2.5 μg L51P implants.

#### 3.2.1 Histological analysis of the femoral repair site

**Fig. 2:**
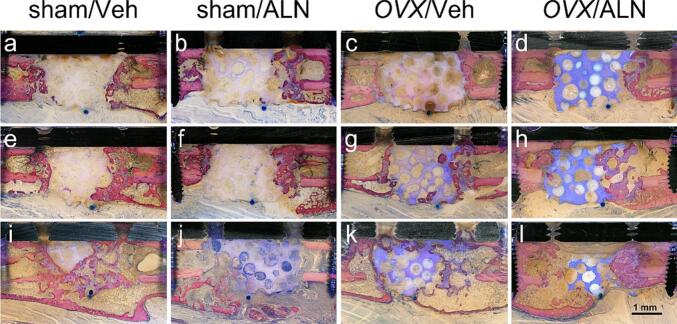
Histological analysis of the femoral repair site six weeks post-implantation. Ground sections were prepared from the methyl methacrylate-embedded repair tissues isolated six weeks after the application of the critical-size defect in sham and *OVX* animals with Veh and ALN therapy. Bone defects were fitted with βTCP-ceramics loaded with 0.25 μg BMP2 (a–d), 0.25 μg BMP2/2.5 μg L51P (e–h), or 2.5 μg BMP2 (i–l). Sections were stained with a mixture of toluidine blue and MacNeal's tetrachrome solution. The coating with 0.25 μg BMP2 did not induce bone healing in sham and *OVX* animals (a–d). The combination of 0.25 μg BMP2/2.5 μg L51P caused minor bone formation coupled with implant removal in the sham and *OVX* groups (e–h). Implants loaded with 2.5 μg BMP2 led to the strongest induction of bone healing in all experimental groups (i–l). The femur is oriented with the distal end on the left side and the proximal part on the right. The scale bar represents 1 mm.

**Fig. 3:**
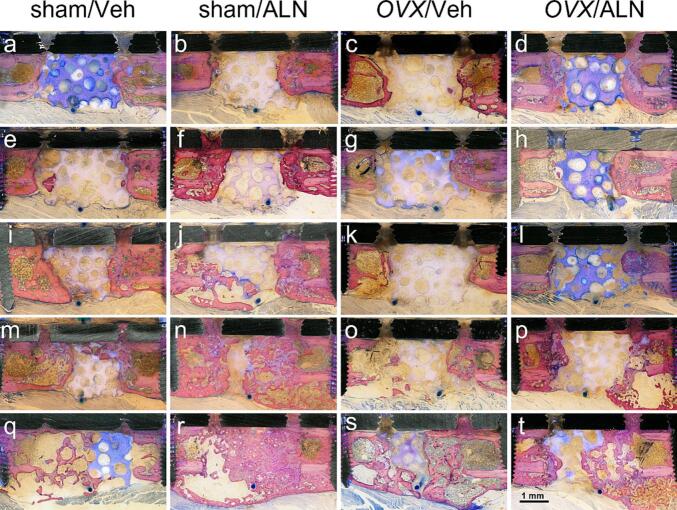
Histological analysis of the femoral repair site twelve weeks post-implantation. Ground sections were prepared from the methyl methacrylate-embedded repair tissues isolated twelve weeks after the application of the critical-size defect in sham and *OVX* animals with Veh and ALN therapy. Bone defects were fitted with unloaded (0 μg BMP2/0 μg L51P) βTCP-ceramics (a–d), or with implants coated with 2.5 μg L51P (e–h), 0.25 μg BMP2 (i–l), 0.25 μg BMP2/2.5 μg L51P (m–p), or 2.5 μg BMP2 (q–t). Sections were stained with a mixture of toluidine blue and MacNeal's tetrachrome solution. Unloaded implants (a–d) and βTCP-ceramics coated with 2.5 μg L51P (e–h) resulted in minimal bone formation in all experimental groups. The coating with 0.25 μg BMP2 induced bone healing in sham animals (i, j), whereas *OVX* groups (k, l) showed minor bone formation. The combination of 0.25 μg BMP2/2.5 μg L51P caused considerable bone formation coupled with implant removal in sham groups (m, n) and induced minimal bone growth in *OVX* animals (o, p). Implants loaded with 2.5 μg BMP2 led to the strongest induction of bone healing in all groups (q–t). The femur is oriented with the distal end on the left side and the proximal part on the right. The scale bar represents 1 mm.

The healing process of the critical-size defect was visualized histologically six (Fig. 2) and twelve (Fig. 3) weeks post-implantation. Bone formation developed from the proximal and distal sides of the bone defect with endosteal and periosteal reactions. The press-fit integration and stable fixation of the βTCP-ceramics promoted the direct ingrowth of bone tissue into the interconnected pores of the graft material.

Six weeks post-surgery, coating with 0.25 μg BMP2 did not induce bone formation within the defect in sham and *OVX* animals, independent of BP therapy (Fig. 2a–d). Twelve weeks post-surgery, 0.25 μg BMP2 induced bone healing from the defect ends in sham animals (Fig. 3i, j), whereas *OVX* groups showed minor bone formation (Fig. 3k, l). Additional control groups at the time point twelve weeks, unloaded (Fig. 3a–d) and 2.5 μg L51P (Fig. 3e–h), displayed minimal bone formation within the defect in all animal groups. In contrast, ceramics loaded with 2.5 μg BMP2 induced bone healing and implant removal independent of estrogen-depletion and BP treatment. Moreover, newly formed bone emanating from the adjacent bone cortex was growing around the βTCP-implant, nearly reaching from the proximal to the distal defect end both six (Fig. 2i–l) and twelve (Fig. 3q–t) weeks post-implantation.

Six weeks post-implantation, the combination of 0.25 μg BMP2/2.5 μg L51P caused minor bone formation, mainly arising from the proximal defect end in sham and *OVX* animals (Fig. 2e–h). Twelve weeks post-implantation, 0.25 μg BMP2/2.5 μg L51P induced minimal bone growth in *OVX* animals (Fig. 3o, p), whereas considerable bone formation coupled with implant removal was detected in sham animals (Fig. 3m, n). In general, the histological analysis showed that only 2.5 μg BMP2 had osteoinductive effects leading to enhanced bone formation within the critical-size defect, whereas all other coatings did not stimulate the healing process.

#### 3.2.2 Histomorphometry of the developing repair tissue

**Fig. 4:**
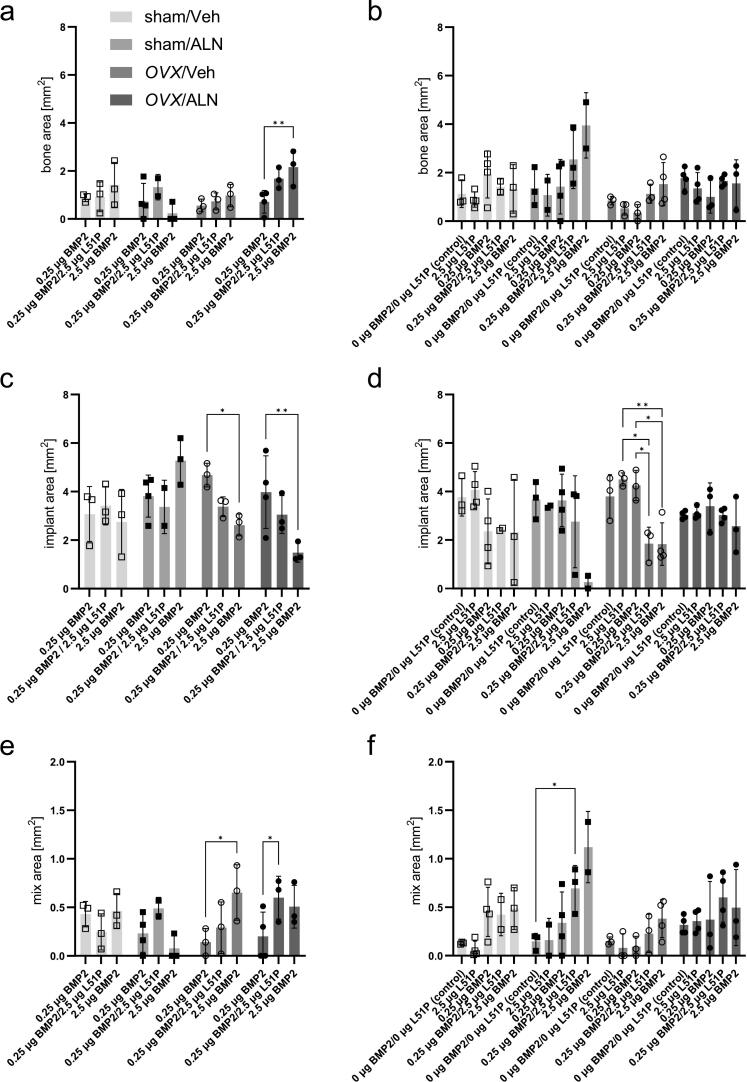
Histomorphometry of newly formed bone and remaining implant material. Histomorphometric analysis of the developing repair tissue was performed by manual selection on one histological section per animal. Assessment of the total bone area (a, b), the remaining implant material (c, d), and mixed tissue comprised of implant material mixed with ingrown bone (e, f) in all groups six and twelve weeks after application of the defect, respectively. Statistical differences were determined within the treatment groups regarding the effect of different implant coatings using a two-way ANOVA with Tukey post-hoc testing. Differences were termed statistically significant with values of *p* ≤ 0.05 (*), *p* ≤ 0.01 (**). Data presented as mean ± standard deviation with 2–4 biological replicates.

Histomorphometric analysis was performed to assess newly formed bone within the defect site (Fig. 4a, b), remaining implant material (Fig. 4c, d), and mixed tissue representing implant material mixed with ingrown bone (Fig. 4e, f). Six weeks post-implantation, significant differences between the 0.25 μg BMP2 group and other coatings were observed only in the *OVX*/ALN group. The exposure to 2.5 μg BMP2 resulted in 3-fold higher bone formation in estrogen-deficient animals under BP therapy (*p* = 0.0069) (Fig. 4a). Twelve weeks post-implantation, no significant differences in the formation of bone were detected within the defect in sham and *OVX* animals, independently of BP therapy or implant coating (Fig. 4b). However, in sham animals under BP treatment, coating with 2.5 μg BMP2 and 0.25 μg BMP2/2.5 μg L51P resulted in 2-fold and 3-fold higher bone formation, respectively, compared to negative controls (unloaded, 2.5 μg L51P, 0.25 μg BMP2). Six weeks post-implantation, coating with 2.5 μg BMP2 significantly reduced the remaining βTCP-material by almost 50 % in *OVX* animals with Veh (*p* < 0.05) and ALN (*p* < 0.01) therapy compared to 0.25 μg BMP2 (Fig. 4c).

Twelve weeks post-implantation, coating with 2.5 μg BMP2 and 0.25 μg BMP2/2.5 μg L51P resulted in an approx. 60 % decrease of the remaining βTCP-implant in *OVX*/Veh animals compared to defects fitted with 2.5 μg L51P or 0.25 μg BMP2 cylinders (p ≤ 0.05) (Fig. 4d). Six weeks post-implantation, the analysis of the tissue fraction comprised of implant material with ingrown bone, revealed a nearly 5-fold increase of the mixed tissue in *OVX*/Veh animals treated with 2.5 μg BMP2 when compared to animals treated with 0.25 μg BMP2 implants (*p* = 0.01). A similar effect was observed in *OVX*/ALN animals, which received 0.25 μg BMP2/2.5 μg L51P implants (*p* < 0.05) (Fig. 4e). Twelve weeks post-implantation, loading with 0.25 μg BMP2/2.5 μg L51P led to 5-fold increase in the volume of mixed tissues in the sham/ALN group compared to animals treated with unloaded controls (*p* < 0.05) (Fig. 4f). The detected amount of mixed tissue correlated with new bone formation and higher implant removal. The histomorphometric analysis showed that the removal of the βTCP-material was directly coupled to the amount of bone formation within the critical-size defect. It further confirmed the osteoinductive effect of implants coated with 2.5 μg BMP2.

#### 3.2.3 MicroCT analysis of the developing repair tissue

MicroCT analysis was performed to assess the volume of the repair tissue (Suppl. Fig. 4a, b), the volume of mineralized tissue consisting of newly formed bone and remaining implant (Suppl. Fig. 4c, d), and the BV/TV of the repair tissue (Suppl. Fig. 4e, f). The average volume of repair tissue remained constant at both time points when animals received unloaded implants or cylinders loaded with 2.5 μg L51P, 0.25 μg BMP2, and 0.25 μg BMP2/2.5 μg L51P. In contrast, exposure to 2.5 μg BMP2 increased the average volume of the repair tissue by approx. 60 % in *OVX*/ALN animals compared to all other coatings at both time points (p < 0.05) (Suppl. Fig. 4a, b). Six weeks post-implantation, 2.5 μg BMP2 led on average to 57.9 ± 34.0 % more mineralized tissue in *OVX*/ALN animals when compared to 0.25 μg BMP2 (*p* < 0.001) (Suppl. Fig. 4c). Twelve weeks post-implantation, 2.5 μg BMP2 resulted in higher amounts of mineralized tissue in all groups, particularly in animals treated with BP (Suppl. Fig. 4d). Six weeks post-implantation, coating with 2.5 μg BMP2 led to approx. 30 % lower BV/TV of the defect tissue in *OVX*/Veh animals in comparison to 0.25 μg BMP2 and 0.25 μg BMP2/2.5 μg L51P (*p* = 0.02) (Suppl. Fig. 4e). Twelve weeks post-implantation, BP therapy increased the BV/TV of the defect tissue independent of the implant coating (Suppl. Fig. 4f). The data demonstrates that 2.5 μg BMP2 stimulated the formation of mineralized tissue, and the amount of repair tissue partially correlated with a higher BV/TV, particularly in combination with BP.

### 3.3 Transcriptome analysis in the developing repair tissues

#### 3.3.1 RNA sequencing

To evaluate the molecular mechanisms regulating bone repair, RNA was isolated from tissues within the defect site at six and twelve weeks after the introduction of the critical-size defect, and a global RNA sequencing analysis was performed. On average, 32.7 million reads/library with an overlap of approx. 86 % (minimum 82 %, maximum 89 %) with the annotated genome (GRCm39, ENSEMBL) were obtained.

#### 3.3.2 Clustering, differentially expressed genes and GO enrichment analysis

**Fig. 5:**
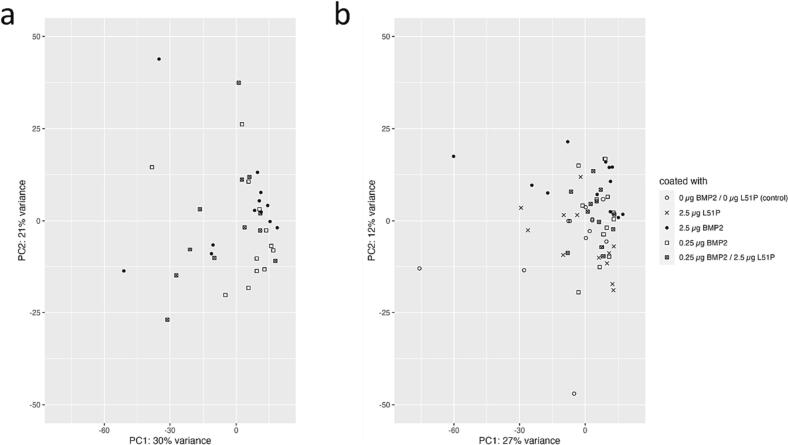
Implant coatings did not result in distinct data clustering. Principal component analysis based on the 500 genes with the most variable expression did not reveal a distinct clustering of the samples taken six (a) weeks or twelve (b) weeks after the application of the critical-size defect in dependence of the bioavailability of BMP2 and L51P.

The first two axes of the principal component analysis, based on the 500 genes with the most variable expression over the whole transcriptome, did not reveal a distinct clustering of the samples in dependence of time point after the application of the critical-size defect, BP therapy, or estrogen-deficiency. In addition, no clustering was observed, when comparing impact of the bioavailability of BMP2 and L51P on the transcriptome at six and twelve weeks post-surgery (Fig. 5).

To further explore the gene expression profile of the developing repair tissues, the numbers of differentially expressed genes (DEG) and the overrepresentation of DEG in the gene ontology (GO) enrichment analysis were assessed. This allows the evaluation of potential changes in the transcriptome linked to bone regeneration with respect to estrogen-deficiency, BP therapy, the effects of BMP2 and L51P, and the time point post-implantation.

##### 3.3.2.1 Exposure to 2.5 μg BMP2 led to a similar transcriptome profile six and twelve weeks post-implantation

Tissue samples harvested six or twelve weeks after MouseFix™ surgery were grouped together regarding the coatings of the respective implants. Focusing on the differences in the temporal evolution of the bone healing process in the presence of BMP2 and L51P between the time points six and twelve weeks post-surgery, the parameters estrogen-depletion and BP therapy were not considered in the analysis (Suppl. Table 1).

The differential gene expression analysis revealed minor changes on the level of the transcriptome in samples with implants coated with 2.5 μg BMP2 with respect to the time after surgery (98 DEG). In contrast, in animals treated with 0.25 μg BMP2/2.5 μg L51P implants, 934 DEG were detected with respect to the time after surgery. Twelve weeks post-surgery, treatment with 0.25 μg BMP2/2.5 μg L51P resulted in the upregulation of biological processes linked to muscle regeneration (GO:0003012 “muscle system process”, GO:0060537 “muscle tissue development”, GO:0014706 “striated muscle tissue development”). In addition, in animals treated with 0.25 μg BMP2 implants, 157 DEG were detected with respect to the time after surgery. Twelve weeks post-surgery, treatment with 0.25 μg BMP2 resulted in the downregulation of biological processes linked to extracellular matrix organization (GO:0030198 “extracellular matrix organization”) and bone repair (GO:0001503 “ossification”, GO:0031214 “biomineral tissue development”, GO:0110148 “biomineralization”, GO:0061448 “connective tissue development”, GO:0051216 “cartilage development”). Six weeks post-surgery unloaded and 2.5 μg L51P implants were not included in the experimental design. Taken together, the comparison of the transcriptome profiles of repair tissues, demonstrates that 2.5 μg BMP2 accelerated the repair process.

##### 3.3.2.2 Exposure to 2.5 μg BMP2 accelerated the bone repair process

Tissue samples harvested six or twelve weeks after MouseFix™ surgery were grouped together regarding the coatings of the respective implants. The parameters estrogen-depletion and BP therapy were not considered in the analysis to detect the impact of the implant coating on the transcriptomic level within the time points post-surgery (Table 1). Six weeks post-surgery, exposure to 2.5 μg BMP2 led to changes in the differential gene expression profile as compared to 0.25 μg BMP2 (145 DEG). The GO enrichment analysis revealed the downregulation of GO terms associated with bone repair (GO:0061448 “connective tissue development”, GO:0048705 “skeletal system morphogenesis”, GO:0060348 “bone development”), cartilage development (GO:0051216 “cartilage development”, GO:0002062 “chondrocyte differentiation”), and extracellular matrix organization (GO:0030198) in animals treated with 2.5 μg BMP2. The comparison between 2.5 μg BMP2 and 0.25 μg BMP2/2.5 μg L51P revealed 3 DEG. Twelve weeks post-surgery, exposure to 2.5 μg BMP2 led to changes in the differential gene expression profile as compared to unloaded controls (1242 DEG) and implants coated with 2.5 μg L51P (1341 DEG), 0.25 μg BMP2 (175 DEG) or 0.25 μg BMP2/2.5 μg L51P (381 DEG). The GO enrichment analysis revealed the downregulation of GO terms associated with extracellular matrix organization (GO:0030198 “extracellular matrix organization”) and bone healing (GO:0061448 “connective tissue development”, GO:0051216 “cartilage development”) in animals treated with 2.5 μg BMP2. No changes on the transcriptome level were detected in the defect tissue between animals that received 0.25 μg BMP2/2.5 μg L51P, 0.25 μg BMP2, 2.5 μg L51P, or unloaded implants. The transcriptome analysis between defect tissues exposed to 2.5 μg BMP2 and all other coatings, further confirmed the conclusion that 2.5 μg BMP2 accelerated the healing process.

**Table 1:** Effect of 2.5 μg BMP2 on the bone healing process at the transcriptome level. Differential gene expression analysis, of the developing repair tissue in animals treated with 2.5 μg BMP2 compared to all other coatings six and twelve weeks post-surgery (*n* = 12).

	2.5 μg BMP2 vs. all other coatings	
	6 weeks	12 weeks
0 μg BMP2/0 μg L51P (control)	–	1242 (331↑, 911↓)
2.5 μg L51P	–	1341 (424↑, 917↓)
0.25 μg BMP2	145 (12↑, 133↓)	175 (54↑, 121↓)
0.25 μg BMP2/2.5 μg L51P	3 (2↑, 1↓)	381 (102↑, 279↓)

##### 3.3.2.3 Estrogen-deficiency induced inflammatory responses within the repair tissue twelve weeks post-surgery

To detect the general effects of *OVX* on the transcriptome profile of the repair tissue six and twelve weeks post-surgery, samples from *OVX* animals were compared to samples from sham animals. Implant coating and BP therapy were not considered in the analysis. Six weeks after the defect, estrogen-deficiency did not cause changes in the differential gene expression analysis. Twelve weeks post-surgery, 378 DEG were detected between the samples from *OVX* and sham animals. *OVX* samples showed upregulation of GO terms linked to B cell mediated immune responses (GO:0019724 “B cell mediated immunity”, GO:0002455 “humoral immune response mediated by circulating immunoglobulin”, GO:0050853 “B cell receptor signaling pathway”). Twelve weeks post-surgery, estrogen-depletion induced inflammatory responses in the developing repair tissue. This resulted in significant changes in the function and regulation of B- and T-cells twelve weeks post-surgery in *OVX* animals.

##### 3.3.2.4 BP therapy altered osteoclast differentiation within the repair tissues six weeks post-surgery

To detect the general effects of BP treatment on the transcriptome profile of the developing repair tissues at time points six and twelve weeks, samples of ALN treated animals were compared to samples from animals receiving Veh only. The implant coating and estrogen-depletion were not considered in the analysis. Six weeks after the application of the defect, 151 DEG were detected within the defect tissues between the samples from Veh and ALN treated animals. The tissues harvested from ALN treated animals showed upregulation of the GO term “multinuclear osteoclast differentiation” (GO:0072674). Twelve weeks after application of the defect, the comparison of the repair tissue revealed only 95 DEG between Veh and ALN treated animals. Six weeks post-surgery, BP therapy altered the function and regulation of osteoclasts in the developing repair tissue.

##### 3.3.2.5 BP therapy led to significant upregulation of selected osteoclast genes

To detect the general effects of BP treatment on the expression of genes essential for osteoclast development and function at time points six and twelve weeks, samples of ALN treated animals were compared to samples from animals receiving Veh only (Table 2). The implant coating and estrogen-depletion were not considered in the analysis. ALN therapy resulted in significantly higher levels of transcripts encoding selected markers of osteoclast lineage cells compared to control animals receiving Veh only. Transcripts encoding osteoclast traits like Oscar, Ocstamp, Dcstamp, Slc9b2, Mmp9, Ctsk, Fos, and Acp5 showed upregulation upon ALN therapy at both time points post-surgery. To visualize the effect of BP therapy on the differential gene expression of the selected osteoclast marker genes, heatmaps were rendered for both time points (Suppl. Fig. 5).

**Table 2:** Differential expression of selected osteoclast genes in the defect site six and twelve weeks post-implantation in ALN-treated compared to Veh-treated animals. Effect of ALN therapy on the expression of transcripts encoding markers of the osteoclast trait during bone healing in critical-size femoral defects at six or twelve weeks after implantation of βTCP-implants. Differential gene analysis was performed by DESeq2 (*n* = 18 for 6 weeks, *n* = 30 for 12 weeks).

	ALN vs. Veh			
	Log 2 fold change		Adjusted p value	
	6 weeks	12 weeks	6 weeks	12 weeks
Ocstamp	1.49	1.77	2.08E−10	1.82E−27
Oscar	1.03	1.34	0.0001	4.05E−14
Dcstamp	1.01	0.91	3.33E−06	1.49E−07
Slc9b2	0.83	1.15	0.0018	1.29E−16
Mmp9	0.79	0.89	1.06E−07	8.03E−13
Ctsk	0.77	0.96	0.0018	4.64E−08
Fos	0.66	0.01	0.0377	0.9903
Acp5	0.60	0.90	0.0130	4.22E−12
Nfatc1	0.49	0.51	3.32E−05	5.72E−15
Tnfrsf11a	0.43	0.46	0.0436	1.05E−05
Slc4a2	0.41	0.44	0.0003	7.13E−11
Src	0.38	0.33	0.0004	0.0008
Calcr	0.35	0.76	0.4313	0.0050
Atp6v1a	0.30	0.37	0.0189	0.0003
Clcn7	0.28	0.30	0.0262	8.19E−06

##### 3.3.2.6 Increased expression levels of osteoclast related genes in the developing repair tissue six weeks post-surgery

To further investigate the temporal evolution of genes essential for osteoclast development and function, samples collected six and twelve weeks after implantation of βTCP-implants were compared either under ALN therapy or Veh treatment (Table 3).

**Table 3:** Temporal evolution of the expression of selected osteoclast genes in the defect site under Veh or ALN treatment. Temporal evolution (6 weeks vs. 12 weeks after implantation of βTCP-ceramics) of the expression of transcripts encoding markers of the osteoclast trait in critical-size femoral defects under ALN therapy or Veh treatment. Analysis performed by DESeq2 (n = 18–30).

	6 weeks vs. 12 weeks			
	Log 2 fold change		Adjusted *p* value	
	Veh	ALN	Veh	ALN
Oscar	0.98	0.46	3.66E−05	0.0959
Ocstamp	0.82	0.33	0.0007	0.1948
Acp5	0.72	0.34	3.23E−05	0.1169
Calcr	0.69	0.50	0.0103	0.2105
Slc9b2	0.68	0.25	6.36E−04	0.3447
Dcstamp	0.49	0.39	0.0571	0.0657
Traf6	0.44	0.07	2.48E−11	0.5540
Mmp9	0.42	0.21	0.0118	0.2875
Tnfrsf11a	0.40	0.30	0.0315	0.0135
Slc4a2	0.38	0.29	0.0002	0.0023
Nfatc1	0.33	0.19	0.0002	0.1010
Clcn7	0.33	0.31	0.0019	0.0004
Atp6v1a	0.31	0.17	0.0090	0.1264
Csf1r	0.26	0.31	0.0829	0.0008
Fos	−0.01	0.57	0.9900	0.0143
Tfrc	−0.23	−0.43	0.5460	0.0391

The implant coating and estrogen-depletion were not considered in the analysis. Levels of transcript of selected osteoclast marker genes (Oscar, Ocstamp, Acp5, Calcr, Slc9b2, Dcstamp) were higher at six weeks as compared to twelve weeks in both Veh and ALN treated animals. The relevance of the respective time point was further emphasized in heatmaps displaying the relative gene expression of the selected osteoclast markers (Suppl. Fig. 6).

##### 3.3.2.7 Increased expression levels of osteoblast related genes in the developing repair tissue six weeks post-surgery

To investigate the temporal evolution of the expression of genes essential for osteoblast development and function, samples from animals treated either with ALN or Veh were collected six and twelve weeks after implantation of βTCP-implants and compared (Table 4). Implant coating and estrogen-depletion were not considered in the analysis. The transcript levels of selected osteoblast marker genes (Col1a1, Cola1a2, Sp7, Bglap, Sparc, Ibsp, Alpl, Tnfsf11, Runx2, Bgn) were higher at six weeks as compared to twelve weeks in both Veh and ALN treated animals. Heatmaps of the relative gene expression of the selected osteoblast marker genes emphasize the relevance of the respective time point (Suppl. Fig. 7).

**Table 4:** Temporal evolution of the expression of selected osteoblast genes in the defect site under Veh or ALN treatment. Temporal evolution (6 weeks vs. 12 weeks after implantation of βTCP-ceramics) of the expression of transcripts encoding markers of the osteoblast trait in critical-size femoral defects under ALN or Veh treatment. Analysis performed by DESeq2 (*n* = 18–30).

	6 weeks vs. 12 weeks			
	Log 2 fold change		Adjusted p value	
	Veh	ALN	Veh	ALN
Col1a1	1.07	1.07	2.88E−06	3.24E−10
Col1a2	1.00	0.96	2.93E−07	7.82E−10
Sp7	0.92	0.44	2.98E−05	0.0324
Sparc	0.84	0.30	1.33E−05	0.2385
Ibsp	0.82	0.77	0.0089	1.29E−07
Alpl	0.70	0.45	0.0044	0.0209
Tnfsf11	0.50	0.34	0.4117	0.3277
Runx2	0.47	0.24	0.0007	0.0263
Bgn	0.42	0.87	0.1736	0.0002
Csf1	0.09	0.33	0.6342	0.0048

## 4 Discussion

Osteoporosis is a major health issue for aging societies and affects hundreds of millions of people worldwide, predominantly postmenopausal women (Willers et al., 2022). Anti-resorptive drugs, in particular BP, are the therapy of choice to prevent bone loss in osteoporosis (Liu et al., 2019; Bone et al., 1997; Cranney et al., 2002; Hosking et al., 1998; Liberman et al., 1995). The consequences of long-term therapies with BP are controversially discussed as the “frozen” bone state and blocked osteoclast activity may impair bone healing and removal of bone grafts (Diab and Watts, 2012; McClung et al., 2013; Watts and Diab, 2010; Odvina et al., 2005; Molvik and Khan, 2015). The present study aimed to investigate the repair process of a critical-size femoral defect, fitted with βTCP-implants, in a murine model for post-menopausal osteoporosis treated with BP with respect to (i) bone repair, (ii) modulation of cellular and chemical resorption of βTCP-implants, and (iii) the effects of BMP2 and L51P on the healing process.

In the present study, estrogen-depletion induced by *OVX* was utilized to generate a murine model for post-menopausal osteoporosis in outbred NMRI mice. The increase in body weight and significant shrinkage of the uterus in *OVX* animals provided evidence of estrogen deprivation. Even though cancellous bone is more sensitive to bone loss induced by estrogen deficiency than is cortical bone, the changes in bone metabolism will be the same over the whole skeleton. MicroCT analysis confirmed the ovariectomy-induced osteopenic bone changes, and BP treatment, starting eight weeks after *OVX*, increased bone mass. Elevated bone density was also detected upon BP therapy in sham controls. BP bind to the bone surface, preferentially at bone sites with increased turnover, affecting bone modeling and remodeling systemically (Sato et al., 1991).

The critical-size defect was applied mid-femur to allow the stable fixation of the defect by the osteosynthesis system. Histological analysis of the critical-size defect six weeks post-implantation demonstrated that 2.5 μg BMP2 accelerated fracture repair and induced bone formation compared to controls (unloaded, 2.5 μg L51P, 0.25 μg BMP2). Binding of 2.5 μg BMP2 to the implant led to new bone formation around the βTCP-implant from the proximal to the distal defect end, irrespective of estrogen-deprivation or BP therapy. Exposure to 0.25 μg BMP2/2.5 μg L51P, however, did not elevate bone formation or promote fracture healing, indicating that L51P did not increase the bioefficacy of BMP2 in the applied defect model.

Efficient bone repair requires specific action of growth factors with precise temporal and spatial distribution (Dumic-Cule et al., 2018; Czech and Oyewumi, 2021). Thus, the bioefficacy of the applied BMP2 is affected by multiple factors beyond the presence of BMP antagonists. Approx. 60 % of the applied BMP2 and L51P are released from the βTCP-ceramics within the first 24 h (Sebald et al., 2012). In cases of large defects, this rapid release may not be sufficient to stimulate bone regeneration (El Bialy et al., 2017). As a result, supraphysiological BMP2 doses only marginally support healing in later repair phases. Additionally, simultaneous administration of L51P and BMP2 may not ideally contribute to the healing process, as the upregulation of BMP antagonists occurs with a chronological delay (Montjovent et al., 2013). The effect of L51P may also depend on the species and age of the applied rodent model, as combined administration of BMP2 and L51P significantly improved fracture healing in retired breeder rats (Hauser et al., 2018b; Sebald et al., 2012).

Both histological and MicroCT evaluation confirmed that new bone formation within the critical-size defect was directly correlated to the removal of the βTCP-implant. This coupling resulted in a comparable volume of total repair tissue among all experimental groups. BP therapy partially enhanced bone healing, leading to elevated levels of repair tissue and higher amounts of mineralized tissue, particularly in animals treated with 2.5 μg BMP2 implants. Overall, in the applied fracture model, the administration of BP did not affect the kinetics of the bone repair process. This observation contrasts with the anticipated outcome, since it was hypothesized that bone formation, through coupling, would be decreased in animals under BP therapy, consequently leading to a delayed healing process compared to animals not subjected to BP treatment. Twelve weeks post-surgery, the implant had not been fully removed, and the critical-size defect had not completely healed, lacking a continuous cortical bone structure or marrow cavity in all experimental conditions. Therefore, distinct differences due to ALN treatment might become detectable later in the healing process only.

The effect of BP on fracture healing remains controversial, with clinical studies showing minor or no influence on bone repair in humans (Begkas et al., 2019; Kates and Ackert-Bicknell, 2016; Gao et al., 2021; Xue et al., 2014). The outcome in rodent models varied from delayed healing to improved mechanical stability of bone defects, depending on the agent, dosage, and treatment protocol (Hadjiargyrou, 2022). In rat fracture models, BP caused delayed unions and reduced healing: in particular, ALN impaired implant removal and increased the callus size (Li et al., 2001; Li et al., 1999; Kidd et al., 2011; Manabe et al., 2012; Cao et al., 2002; Fu et al., 2013; Hauser et al., 2018b). Species-dependent differences and characteristics of the applied model, including the age of the animals and the type of fracture, might explain the discrepancy in the impact of BP on bone regeneration (Hadjiargyrou, 2022). Independently of the cellular resorption, the degradation of the βTCP implant might be partially attributable to a chemical dissolution process due to a decrease of the pH levels within the healing defect. This process can be initiated by a mechanism called intrinsic osteoinduction caused by the implantation of the βTCP-material itself (Bohner et al., 2020; Bohner and Miron, 2019). The implanted graft acts as a nucleation site for the precipitation of carbonated apatite resulting locally in lower concentrations of phosphate and calcium ions. This biomimetic apatite layer stimulates the differentiation of osteoblast precursor and stem cells initiating bone formation linked to the gradual breakdown of the implant material (Bohner et al., 2020; Bohner and Miron, 2019).

In the present study, transcriptome analysis of the repair tissue showed no data clustering due to the extensive variables in the experimental setup. The severe intervention of creating the femoral defect activated intrinsic healing processes, outweighing the effects of estrogen-deficiency and BP therapy, as seen in previous studies (Hauser et al., 2018a). In that study, the mode of fixation was found to have the strongest impact on the healing process. Rigid compared to non-rigid fixation of femoral osteotomies initiated different developmental programs, endochondral healing compared to direct bone formation, respectively. However, other parameters did not significantly differentiate the experimental groups (Hauser et al., 2018a).

Changes on the level of transcription related to implant coating were detectable when implants were coated with 2.5 μg BMP2. Differential gene expression analysis indicated accelerated repair, and GO terms associated with bone healing and tissue regeneration were downregulated twelve weeks post-surgery. This finding aligns with the histological analysis, showing significant bone formation already six weeks after defect treatment with 2.5 μg BMP2. The temporal evolution of selected osteoclast and osteoblast genes showed higher expression levels at six weeks compared to twelve weeks. This indicates a more dynamic repair process at six weeks, with prominent cellular responses from both cell lineages. In addition, transcriptome analysis suggested that bone regeneration was almost complete, or the healing capacity was already exhausted six weeks after surgery. The strongest causative factor affecting bone regeneration and the composition of the transcriptome was implant coating, specifically the presence of 2.5 μg BMP2, followed by BP therapy and estrogen-depletion.

The efficacy of the BP therapy was also confirmed on the transcriptome level. Transcripts encoding osteoclast lineage markers at both six and twelve weeks post-surgery showed increased expression levels in the presence of BP, regardless of estrogen-depletion and implant coating. As BP therapy causes osteoclast inactivity (Rodan and Fleisch, 1996; Russell, 2006), the lack of a feedback mechanism that is required to control osteoclastogenesis leads to the continuous recruitment of osteoclasts to the repair site (Weinstein et al., 2009). An increase in the recruitment of osteoclast precursors and enhanced fusion of progenitors forming giant osteoclasts may also contribute to the high expression levels of transcripts encoding the osteoclast marker genes (Hauser et al., 2018a).

Nevertheless, bone formation within the defect stayed linked to implant resorption, further indicating the chemical dissolution of the βTCP-graft due to pH changes in areas with active bone healing. The metabolic activity of osteoblasts might cause the acidification of the microenvironment, facilitating the dissolution of the implanted graft. Moreover, recruited osteoclasts might release factors like Sphingosine-1-phosphate stimulating osteoblast activity (Kim et al., 2020), indirectly fostering the osteogenic environment to promote the healing process, and coupled implant removal.

The present study investigated two key aspects in reconstructive orthopedic surgery. Firstly, it demonstrated that removal of βTCP-ceramics from a repair site can also occur under BP treatment. The osteogenic environment within a defect undergoing repair might initiate the dissolution of the calcium phosphate-based material outweighing the action of the BP therapy. This observation corroborates the results in humans, where the influence of long-term BP treatment on bone repair was minor or absent. Secondly, application of supraphysiological doses of BMP2 remained superior in promoting bone formation. In the used fracture model, L51P did not enhance the bioefficacy of BMP2 when administered simultaneously on βTCP-grafts. As various factors influence the bone repair process, it is not excluded that an adjustment of the applied L51P/BMP2 doses might result in the desired osteoinduction. Thus, further studies would be necessary to investigate the efficacy of L51P in vivo and to discover the optimal dosage and timing of L51P application to enhance bone regeneration. There are, however, also limitations of the utilized fracture model. The developing repair tissue was only examined six and twelve weeks post-surgery and no biomechanical testing of the partially healed defects could be performed. Despite these limitations, however, the data does not provide evidence that BP therapy does impair the healing process of large bone defects treated with βTCP-implants.

## CRediT authorship contribution statement

**Franziska Strunz:** Data curation, Formal analysis, Investigation, Project administration, Writing – original draft, Writing – review & editing. **Saskia Gentil-Perret:** Data curation, Formal analysis, Visualization, Writing – review & editing. **Mark Siegrist:** Data curation, Investigation, Project administration. **Marc Bohner:** Resources, Writing – review & editing. **Nikola Saulacic:** Investigation, Methodology, Writing – review & editing. **Willy Hofstetter:** Conceptualization, Funding acquisition, Methodology, Project administration, Resources, Supervision, Validation, Writing – review & editing.

## Declaration of competing interest

None.

## Data Availability

Data will be made available on request.

## Appendix A Supplementary data

Supplementary material Image 1
