# Supplementary material for: Bisphosphonates do not affect healing of a critical-size defect in estrogen-deficient mice
Source: Bone Rep. 2024 Jan 17;20:101739. doi: 10.1016/j.bonr.2024.101739 (PMC10831175; doi:10.1016/j.bonr.2024.101739)
Supplement: Supplementary file 1 — Supplementary material [file mmc1.docx]

# Supplement

**Suppl. Table 1.** **Temporal evolution of the transcriptome for each coating condition (comparison 12 weeks vs. 6 weeks post-implantation).**

Differential gene expression analysis, of the repair tissue from animals treated with similarly coated βTCP-ceramics, comparison between twelve and six weeks post-surgery (n=12).

|  | **12 weeks vs. 6 weeks** |
| --- | --- |
| **0.25μg BMP2** | 157 (76↑, 81↓) |
| **0.25μg BMP2 / 2.5μg L51P** | 934 (655↑, 279↓) |
| **2.5μg BMP2** | 98 (57↑, 41↓) |


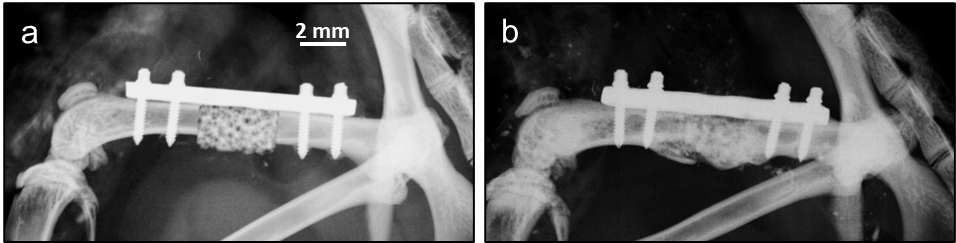


**Suppl. Fig. 1. X-ray imaging for the confirmation of implant placing and fixation.**

Implant placement and fixation with the osteosynthesis system consisting of a titanium plate and four interlocking screws post-surgery (a) and 12 weeks later (b) by X-ray imaging.

**Suppl. Fig. 2. Body weight and uterus dry weight of *OVX* and sham animals.**

Body weight was measured during *OVX*/sham surgery (-13 weeks), application of the critical-size femoral defect (time point 0) and 6 and 12 weeks later (a). Uteri were collected at the endpoint of the study and dried overnight (b). *OVX* animals showed a significant increase in body weight at time point 0 and later. The uterus dry weight was significantly lower in *OVX* animals due to shrinkage after estrogen-depletion. Statistical differences were determined using an unpaired t-test. Data presented as mean with upper and lower quartile and minimum and maximum values (n=55−115, body weight; n=115−121, uterus dry weight).

**Suppl. Fig. 3. MicroCT analyses of lumbar vertebrae 4 (L4).**

Bone volume/total volume (BV/TV), and three-dimensional structure of the vertebral bodies of L4 of sham and *OVX* animals with or without ALN therapy. Data displays the vertebral structure representative for animals six and 12 weeks after the application of the critical-size defect. The BV/TV is significantly lower in *OVX*/Veh animals compared to sham/Veh. ALN therapy led to a significant increase in BV/TV in sham and *OVX* animals, but still, a significant difference between the sham/ALN and *OVX*/ALN group was detected. Statistical differences were determined using a one-way ANOVA with Tukey post-hoc testing. Differences were termed statistically significant with values of p ≤ 0.05. Data presented as mean ± standard deviation with 11−19 biological replicates.

**Suppl. Fig. 4. MicroCT analysis of the developing repair tissue.**

MicroCT analysis of the developing repair tissue was performed by manual selection. Assessment of the volume of the repair tissue (a,b), volume of the mineralized tissue consisting of newly formed bone and the remaining implant material (c,d), and the BV/TV of the repair tissue (e,f) in all treatment groups six and 12 weeks after application of the critical-size defect, respectively. Statistical differences were determined within the treatment groups regarding the effect of different implant coatings using a two-way ANOVA with Tukey post-hoc testing. Differences were termed statistically significant with values of p≤0.05 (*), p≤0.01 (**). Data is presented as the mean ± standard deviation with 2−5 biological replicates.

**
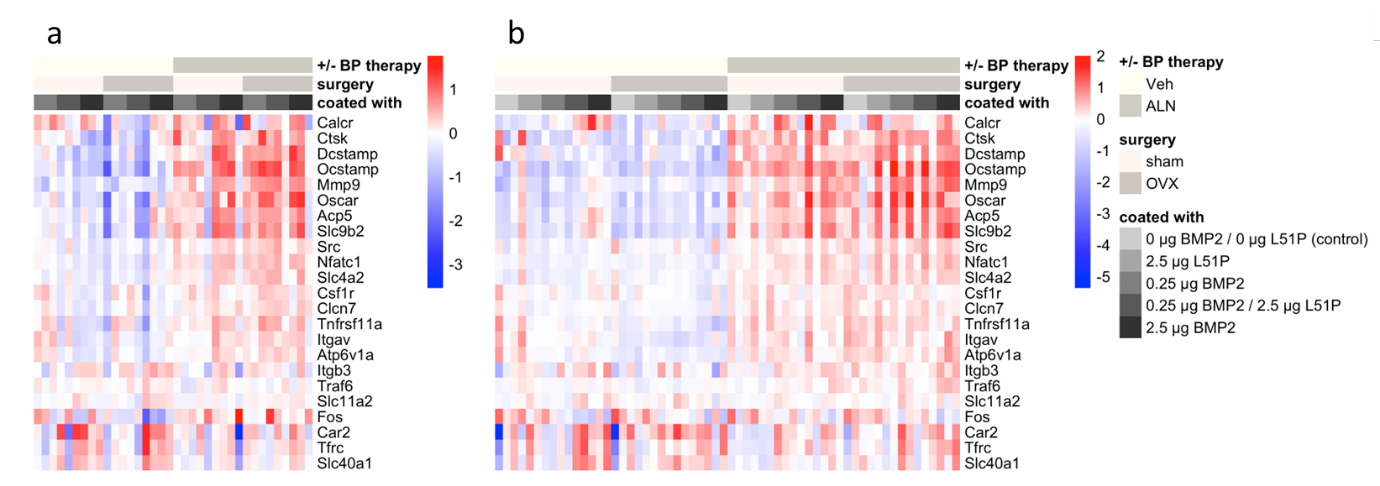
**

**Suppl. Fig. 5. Expression of selected osteoclast marker genes in the defect site six weeks and twelve weeks post-implantation.**

Heatmaps representing the differential gene expression of selected osteoclast markers in the defect tissue harvested six (a) and twelve (b) weeks after application of the critical-size defect. ALN therapy results in higher expression levels of osteoclast markers as compared to animals receiving Veh. Each treatment group consists of 3 biological replicates.


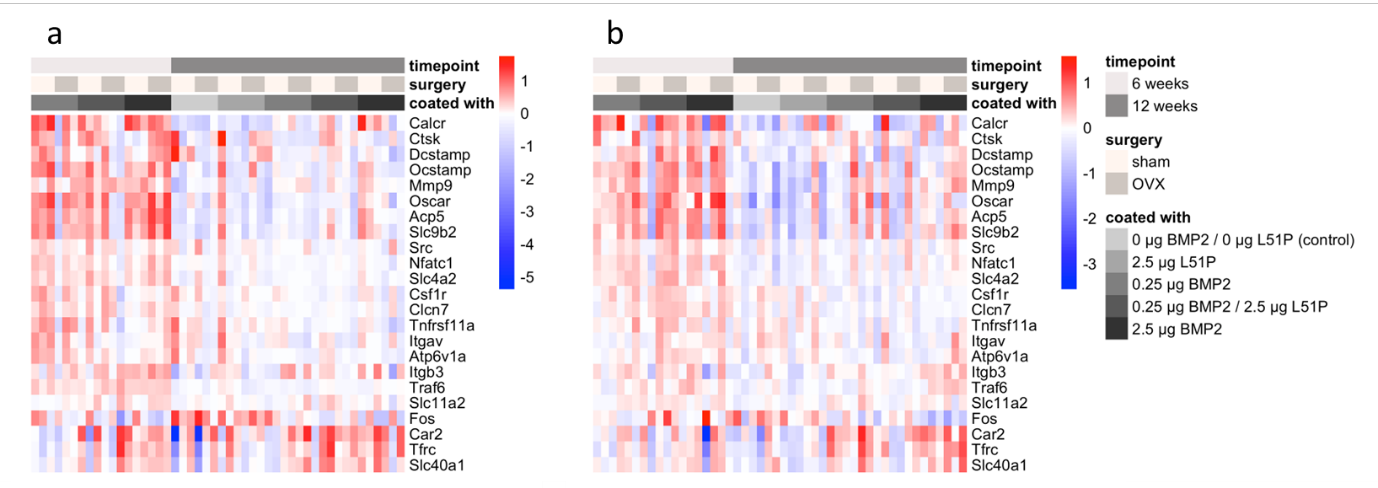


**Suppl. Fig. 6. Temporal evolution of the expression of selected osteoclast marker genes in the defect site under Veh or ALN treatment.**

Heatmaps representing the differential gene expression of selected osteoclast markers in the defect tissue harvested six and 12 weeks after application of the critical-size defect with Veh (a) and BP (b) treatment. Higher expression levels of osteoclast marker genes at six weeks compared to twelve weeks. Each treatment group consists of 3 biological replicates.

**
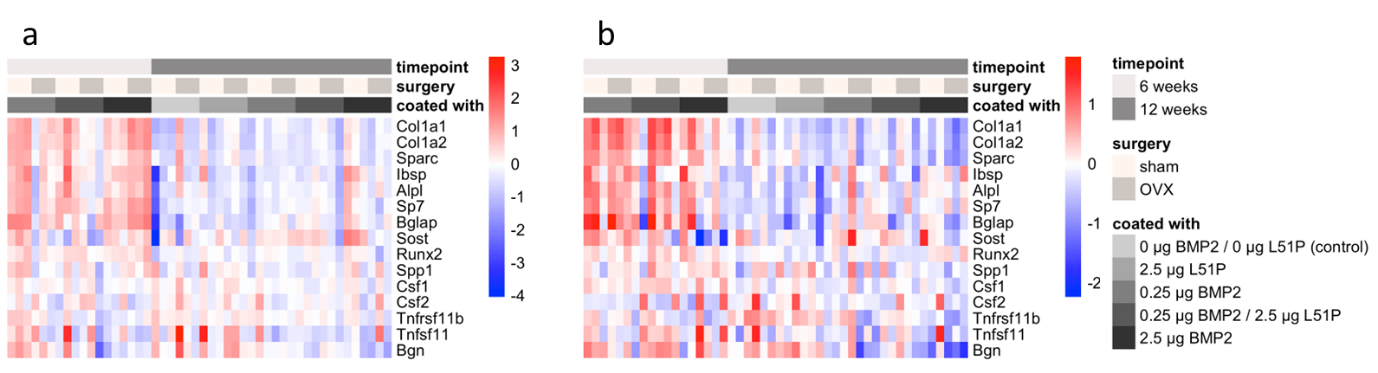
**

**Suppl. Fig. 7. Temporal evolution of the expression of selected osteoblast marker genes in the defect site under Veh or ALN treatment.**

Heatmaps representing the differential gene expression of selected osteoblast markers in the defect tissue harvested six and twelve weeks after application of the critical-size defect with Veh (a) or BP (b) treatment. Higher expression levels of osteoblast marker genes at six weeks compared to twelve weeks. Each treatment group consists of 3 biological replicates.
